# Physicians’ perspectives regarding non-medical switching of prescription medications: Results of an internet e-survey

**DOI:** 10.1371/journal.pone.0225867

**Published:** 2020-01-10

**Authors:** Tabassum Salam, Amy Duhig, Aarti A. Patel, Ann Cameron, Jennifer Voelker, Brahim Bookhart, Craig I. Coleman

**Affiliations:** 1 Medical Education, American College of Physicians, Philadelphia, PA, United States of America; 2 Consulting Services, Xcenda, Palm Harbor, FL, United States of America; 3 Janssen Scientific Affairs, LLC, Titusville, NJ, PA, United States of America; 4 University of Connecticut School of Pharmacy, Storrs, CT, United States of America; 5 Evidence-Based Practice Center, Hartford Hospital, Hartford, CT, United States of America; East Carolina University Brody School of Medicine, UNITED STATES

## Abstract

**Background:**

Physicians are in an ideal position to describe the impact of medication non-medical switching (switching commonly due to formulary changes by insurer for reasons unrelated to patient health) on their practice dynamics and patient care. We sought to examine physicians’ openness to requests for non-medical switching and their experiences and opinions regarding the impact of non-medical switching on their practice, staff and patients.

**Methods:**

An online survey of randomly-sampled physicians spending ≥10% of time providing patient care and having received ≥1 non-medical switch request during the prior 12-months. The impact of non-medical switching on clinical decision-making process; professional experience with clinical practice, patient-physician relationship, insurance process; and perceived impact on practice, staff and patients were assessed. Weighted percent responses were calculated.

**Results:**

We sampled 1,010 physicians (response rate = 55.5%). Many responded being frequently not amenable (26.0%) or had reservations (41.8%) to non-medical switch requests; with >50% indicating patient stability on current therapy and suboptimal alternatives as factors frequently influencing amenability. Physicians agreed non-medical switching can create ethical concerns (clinical judgement, autonomy, ability to treat per guidelines; 74.8%, 82.3%, 53.5%, respectively), while forcing them to take responsibility for insurers’ decisions (81.1%) and diverting their clinical time (84.3%). Most indicated non-medical switching increased practice burden (administrative, non-billable interactions, additional staffing, non-office patient contact, calls to/from the pharmacy; 85.0%, 72.5%, 62.2%, 64.2%, 69.5%, respectively). Physicians felt insurer processes discouraged non-medical switch challenges (76.7%) and required inconvenient lengths-of-time (76.1%) speaking to insurer representatives without proper expertise (62.0%). They believed non-medical switching negatively impacted aspects of care (effectiveness, side-effects, medication adherence and abandonment, out-of-pocket costs, medication errors; 46.5%, 53.2%, 50.6%, 49.4%, 59.6%, 54.5%, respectively).

**Conclusions:**

Physicians were frequently not amenable or had reservations regarding non-medical switching. They noted ethical concerns due to non-medical switching. Most felt non-medical switches burdened their practice and negatively impacted care.

## Introduction

Non-medical switching is typically defined as a change in a stable patient’s prescribed medication to a clinically distinct, non-generic, alternative for reasons other than lack of clinical response, side effects, or poor adherence [1–2]. Non-medical switch requests most often occur due to formulary changes implemented by an insurance company in order to lower their acquisition cost for prescription medications [1–2].

Due to the interplay between patients, physician practices, pharmacies and insurance companies, the process by which a patient and their physician need to respond to a non-medical switch request (either accepting or challenging it) to assure adequate and uninterrupted treatment becomes complex and time consuming (***S1 Fig***). This complex process misaligns with the widely accepted goal—The Quadruple Aim—of enhancing patient experience, improving population health, reducing costs and improving the work life of healthcare providers, including physicians and staff [3–5]. Non-medical switching, while decreasing acquisition costs for prescription medications, has been associated with unintended negative consequences on patients’ clinical outcomes, healthcare utilization and medication adherence/persistence, physician-patient relationship, practice burden and physician and staff morale [6–13]. As a result, non-medical switching has been addressed in guidance documents and position statements released by the American Medical Association (AMA) as well as other medical societies, government entities and patient advocacy groups [1–2, 4–5, 8–13].

Physicians have first-hand experience with the impact of non-medical switching on both their own practice dynamics and patient care. Therefore, we sought to examine the openness of physicians to requests for non-medical switching of pharmaceutical medications, as well as their experiences, opinions and perceptions of the impact of non-medical switching has on their clinical decision making, practice, office staff and patients they care for.

## Materials and methods

### Survey development and testing

The present study used a cross-sectional survey design. The initial version of the survey was developed by investigators after conducting exploratory interviews and a literature search for non-medical switching studies and existing guideline/policy statements. After the draft survey instrument was developed, we conducted 90-minute web-assisted pre-test interviews with 5 screened-in physicians (3 primary care providers and 2 specialists) for further refinement. These physicians were asked questions about the content of the survey (clarity, response options, relevance and comprehensiveness). These physicians were compensated at fair market value rates for 90 minutes of their time and survey feedback. The qualitative interviews were not conducted for the purpose of content validation as survey questions were not developed with the intention to measure specific constructs via an instrument. As such, content validity ratios or indices and Cronbach’s alpha (internal consistency) were not calculated for this study. The final survey included questions addressing 3 general (non-disease or medication specific) physician perspective domains on non-medical switching including: 1) clinical decision-making process (amenability and factors influencing amenability to the change and frequency and success of challenges); 2) professional experience with clinical practice, patient-physician relationship and insurance process; and 3) perceived impact on practice, staff and patients (effect on office administration, staffing, workflow, morale and patient outcomes including access to medication, health status, healthcare utilization and confidence in physician care). An additional set of questions were administered to collect data regarding physicians’ demographics and professional background. Only one survey question (or question block) was displayed per screen; resulting in a maximum of 58 unique screens for the on-line survey (including survey instructions and follow-up questions). Participants were allowed to change their responses until they submitted the survey. The survey (***S1 Appendix*)** was programmed into an online survey platform, Decipher (FocusVision, New York, New York, USA) where internal link testing and testing in a small sample of respondents (≤10) were performed prior to full deployment. Pathways were tested to ensure participant links were correctly directing them to the survey and that those who completed the screener versus those who terminated were properly directed to the registration or termination screen.

### Sample and survey administration

A double “opt-in” (consent to participate) process was utilized for this internet e-survey. Physician participants were identified and recruited by Research Now-SSI (RN-SSI), a global market research firm that has a voluntary US panel. RN-SSI’s physicians have been recruited into their panel via state licensing data and professional associations including the AMA. Participating physicians maintain the opportunity to opt-in to the panel (ie, consent to be contacted for study opportunities). In the present study, a sample of physicians from the RN-SSI panel approximately equally represented across US census geographic regions were randomly selected and sent an email invitation to opt-in to participate in this specific survey. Because participants had already consented to participate in the RN-SSI physician panel and also opted-in to the complete the survey, the need for an additional consent form was waived by an independent institutional review board (IRB) (Solutions IRB, Yarnell, AZ, USA, protocol identification number: 2018/10/8). The purpose of the study was clearly stated in the invitation email. Participants were first required to complete screener questions in order to determine whether they met the present study’s inclusion criteria including being a licensed physician currently practicing (post-residency and/or fellowship >2 years but <30 years) in a general practice, internal medicine, family medicine or specialist setting; spending a minimum of 10% of their professional time providing direct patient care and had received a request for a non-medical switch for at least 1 patient in prior 12-months. All eligible participants based on the screener questions were invited to complete the online survey. Recruitment quotas were set to meet a total *a priori* determined sample size of 1,000 primary care and specialist physicians (based on a prior AMA survey study of physicians regarding prior authorization) [12]. A sampling ratio of 60% primary care/40% specialist physicians was chosen (despite primary care physicians making up less than one-third of active physicians) because of the ~990 million ambulatory care visits Americans undergo annually, 51% are with primary care physicians [13].

Participants were informed the online survey would take approximately 15–20 minutes to complete and physicians received an honorarium of $35 (primary care) or $45 (specialists) upon completion. The survey was fielded from November to December 2018 and remained active until sample size goals were reached. The online survey was designed in such a manner to reduce missing responses, biases (randomization of item responses) and respondent burden (adaptive questioning/skipping to reduce the number and complexity of questions). To ensure the integrity of our data collection, at the end of the survey each respondent was asked to select only the numbers “2” and “8” from a listing of numbers ranging 1–10; with a failure to do so resulting in respondents’ exclusion from the results.

Investigators were blinded to all respondents in order to remain compliant with the Health Insurance Portability and Accountability Act (HIPAA) and participants were blinded to the study sponsor (Janssen Pharmaceuticals, Titusville, NJ, USA) (i.e., double blinding was performed). Only RN-SSI had access to identifying information. This data was stored on a secure system and was not provided in any form to study investigators. Each respondent was assigned a unique ID for data analysis purposes. Investigators did not have access to any participant identifying information other than their basic demographic information collected as part of the survey. This study was approved by the IRB and reporting of results follow the Checklist for Reporting Results of Internet E-Surveys (CHERRIES)[14].

### Data storage

Xcenda hosted the survey and developed a Microsoft Excel (Microsoft Corp., Redmond, WA, USA)-based compendium of results. This electronic data file did not include any respondent-identifying information and was provided to the study sponsor at the completion of the study. Data are stored on a secure Microsoft Sharepoint (Microsoft Corp., Redmond, WA, USA) data management and storage system and will be maintained in accordance with applicable data retention polices.

### Data analysis

The analyses for all study objectives were descriptive in nature. Categorical variables were summarized as counts and percentages, and continuous measures as means with standard deviations (SDs) and medians with ranges. Survey questions were framed as either 5-point (“Very Frequently”, “Frequently”, “Occasionally”, “Rarely”, “Never”) or 7-point (“Increases Greatly”, “Increases Very Much”, “Increases Somewhat”, “No Change”, “Decreases Greatly”, “Decreases Very Much”, “Decreases Somewhat” or “Agree Strongly”, “Agree Very Much”, “Agree Somewhat”, “Neither Agree Nor Disagree”, “Disagree Strongly”, “Disagree Very Much”, “Disagree Somewhat”) ordinal Likert scales. For each survey question, we collapsed the native ordinal Likert scale response into 2 (if no neutral response was possible) or 3 (when a neutral response was possible) level categorical responses by merging responses at the highest and lowest ends of Likert scales in aid in the interpretability of our results. We constructed study specific post-stratification weights to adjust for differential sampling rates and nonresponse by physician type (primary care, specialist) [15–16]. Weighted percentages of physician responses with accompanying 95% confidence intervals (CIs) were reported separately for each question. No missing data were imputed. Data management and analysis were conducted using SAS version 9.4 (SAS Institute, Cary, NC, USA) and STATA version 12 (StataCorp LLC, College Station, TX, USA).

## Results

### Response rate and physician and practice characteristics

In total 21,493 physicians from the RN-SSI panel were available for recruitment into this survey study. Email invitations to opt-in (consent) were sent to a random sample of 13,117 physicians of which 1,904 opened the email and clicked on the embedded survey link (**S1 Table**). Of these physicians who opted-in, 1,032 completed the screener without terminating due to disqualification and were thus eligible for study inclusion. One primary care physician found eligible after completing the screener declined further participation and an additional 22 physician responses were considered invalid (on-line data integrity question failed) and excluded from analysis. Thus, our study included responses from 1,010 physicians (n = 606 primary care; specialists n = 404) who treated patients experiencing non-medical switching (response rate [14–15], 7.7% of all physicians sent an email, 53.0% of physicians receiving an email invitation and clicking on the embedded link). The survey completion rate was 85.7%, with a median time to complete the survey of ~17 minutes.

Demographic and physician practice characteristics are summarized in **Table 1**. The mean age of respondents was 49.6±9.1 years and 68.8% were men. Almost 80% had been in practice for 11 or more years and 97.3% of respondents spent ≥75% of their time providing direct patient care. Over one-half (59.9%) of participating physicians were in private practice. Physicians reported most patients they cared for were covered, to some extent through Medicaid, Medicare/Medicare Advantage or commercial insurance. The most common reimbursement mechanisms for patient care compensation reported were salary (53.1%) and fee-for-service (49.3%).

**Table 1:** Characteristics of respondents.

Characteristic		Overall N = 1,010	
		No.	%
Gender	Female	285	28.2
	Male	695	68.8
	Missing	30	3.0
Age	Mean (SD)	49.55	9.1
Practice Type	General practice	59	5.8
	Internal medicine	334	33.1
	Family medicine	213	21.1
	Specialist	404	40.0
Primary Specialty	Cardiology	81	20.1
	Dermatology	40	9.9
	Endocrinology	81	20.1
	Gastroenterology	40	9.9
	Oncology	40	9.9
	Psychiatry	81	20.1
	Rheumatology	41	10.2
Census Region	Northeast	264	26.1
	Midwest	217	21.5
	South	313	31.0
	West	216	21.4
Years of Practice	<10 years	205	20.3
	11 to 19 years	417	41.3
	20 to 29 years	388	38.4
Proportion of Time Providing Direct Patient Care	10 to 24%	2	0.2
	25 to 49%	4	0.4
	50 to 74%	21	2.1
	≥75%	983	97.3
Practice Setting	Academic/teaching hospital	208	20.6
	Outpatient centers (hospital affiliated)	199	19.7
	Community hospital	219	21.7
	Private practice	605	59.9
	Ambulatory surgical center	16	1.6
	Other	22	2.2
Characteristics of Private Practitioners	Solo practice	144	23.8
	Partnership (2 physicians)	81	13.4
	Same-specialty group practice (3 or more physicians)	210	34.7
	Multi-specialty group practice (3 or more physicians)	170	28.1
Proportion of Time Covered By	Medicaid		
	<25%	712	74.9
	25 to 49%	190	20.0
	50 to 74%	36	3.8
	≥75%	13	1.4
	Medicare/Medicare Advantage		
	<25%	296	31.1
	25 to 49%	482	50.7
	50 to 74%	157	16.5
	≥75%	16	1.7
	Commercial		
	<25%	121	12.7
	25 to 49%	344	36.2
	50 to 74%	366	38.5
	≥75%	120	12.6
	Uninsured and unable to pay		
	<25%	923	97.1
	25 to 49%	25	2.6
	50 to 74%	2	0.2
	≥75%	1	0.1
	Don't know	59	5.8
Reimbursement Mechanisms	Fee-for-service	498	49.3
	Value-based payment arrangements (ie, pay-for-performance, shared savings)	298	29.5
	Salary	536	53.1
	Other	11	1.1

### Domain: Clinical decision-making process

#### Openness to requests/challenging non-medical switches

Nearly 73% of responding physicians stated they were infrequently or not amenable to making a non-medical switch of a prescription medication, without any reservation (**Table 2**). Moreover, only 41.8% of physicians indicated they were frequently amenable to changing a medication, with reservation; while 41.9% were frequently reluctant to changing a medication but opted not to challenge the non-medical switch request due to the insurance appeal process. An additional 26% of physicians noted they were frequently not amenable to the non-medical switch and would contact the insurer to contest the change.

**Table 2:** Clinical decision-making process related to non-medical switching over the last 12 months.

When thinking about your decision regarding a non-medical switch, how frequently are you:	% of Physicians Surveyed Responding “Very Frequently” or “Frequently” (95%CI)^a^	% of Physicians Surveyed Responding “Occasionally”, “Rarely” or “Never” (95%CI)^a^
Amenable to changing the medication without reservation	27.3 (23.6–31.2)	72.7 (68.8–76.4)
Amenable to changing the medication with reservation	41.8 (37.6–46.1)	58.2 (53.9–62.4)
Not amenable to changing the medication, and contact the insurance company to challenge the non-medical switch	26.0 (22.5–30.0)	74.0 (70.0–77.5)
Reluctant to change the medication, but do not challenge the non-medical switch due to the insurance process	41.9 (37.6–46.1)	58.1 (53.9–62.4)
**How often do the following factors influence whether or not you are amenable to a non-medical switch request?**		
Efficacy of the alternative medication	59.6 (55.2–63.7)	40.4 (36.3–44.8)
Side effects of the alternative medication	55.8 (51.5–60.1)	44.2 (39.9–48.5)
Patient’s stability on current medication	72.7 (68.8–76.4)	27.3 (23.6–31.2)
Your prescribing experience with both the current and alternative medication	60.3 (56.0–64.4)	39.7 (35.6–44.0)
Patient concerns about alternative medication	48.4 (44.2–52.8)	51.6 (47.2–55.8)
Patient requests a challenge to the non-medical switch	35.7 (31.6–39.8)	64.3 (60.2–68.4)
Switch would be suboptimal to the patient	50.6 (46.3–54.9)	49.4 (45.1–53.7)
Administrative time required	49.8 (45.5–54.1)	50.2 (45.9–54.4)
Narrow therapeutic window of the medication (eg, warfarin or Synthroid)	35.8 (31.8–40.0)	64.2 (60.0–68.2)
Evidence-based medicine (EBM)/ guidelines	47.3 (43.0–51.6)	52.7 (48.4–57.0)
Frequency of dosing (eg, once daily vs. twice daily)	41.0 (36.9–45.4)	59.0 (54.6–63.1)
Vulnerable populations (eg, minority, elderly, lower socioeconomic income)	44.8 (40.5–49.1)	55.2 (50.9–59.5)
Your past experiences attempting to challenge a non-medical switch with insurance companies in general	49.1 (44.7–53.3)	50.9 (46.7–55.3)
**How often do you challenge a non-medical switch?**		
On average, how often do you challenge a non-medical switch	26.3 (22.6–30.2)	73.7 (69.8–77.4)

^a^All percentages are weighted.

#### Factors influencing openness

The factors that most often influenced whether physicians were amenable to non-medical switch requests (based on responses of “very frequently/frequently” from at least 50% of surveyed physicians) were a patient’s stability on current medication (72.7%), their prescribing experience with both the current and alternative medication (60.3%), efficacy of the alternative medication (59.6%), side effects associated with the alternative medication (55.8%) and physicians’ belief that the non-medical switch would be suboptimal for the patient (50.6%). A small fraction of physicians indicated they frequently challenged a non-medical switch request.

### Domain: Physician opinions

#### Impact on professional clinical practice

Physicians agreed that non-medical switching frustrates them (87.6%), forces them to compromise their ethics (49.2%), compromises their autonomy in clinical decision making (82.3%), undermines their clinical judgment (74.8%), results in treatment inconsistent with accepted guidelines (53.5%) and forces them to take responsibility for a medication decision made by the insurance company (81.1%) (**Table 3**). Nearly two-thirds (65.3%) of physicians disagreed to some extent that a non-medical switch reduces the role of insurers in the medication selection process.

**Table 3:** Physician opinion(s) regarding non-medical switching.

Please indicate how much you agree or disagree with each of the following related to non-medical switching and your professional experience	% of Physicians Surveyed Responding “Agree Strongly”, “Agree Very Much” or “Agree Somewhat” (95%CI)^a^	% of Physicians Surveyed Responding “Neither Agree Nor Disagree” (95%CI)^a^	% of Physicians Surveyed Responding “Disagree Strongly”, “Disagree Very Much” or “Disagree Somewhat” (95%CI)^a^
Frustrates me	87.6 (84.4–90.1)	9.6 (7.5–12.6)	2.8 (1.6–4.5)
Forces me to compromise my ethics	49.1 (44.9–53.5)	32.9 (29.0–37.1)	18.0 (14.8–21.4)
Puts me in an uncomfortable situation where I am conflicted between the patient’s needs and the fiscal responsibilities of my practice	69.8 (65.7–73.7)	20.9 (17.5–24.5)	9.3 (7.1–12.2)
Compromises my autonomy in clinical decision making	82.3 (78.8–85.4)	11.6 (9.2–14.7)	6.1 (4.3–8.4)
Undermines my clinical judgment	74.8 (71.0–78.5)	16.8 (13.8–20.2)	8.4 (6.3–11.1)
Diverts communication time with patients away from other important clinical issues	84.3 (80.8–87.1)	12.1 (9.5–15.2)	3.9 (2.5–5.9)
Undermines a patient’s trust in my ability as a clinician	56.1 (51.7–60.3)	30.8 (26.9–34.9)	13.1 (10.4–16.2)
Results in treatment inconsistent with accepted guidelines	53.5 (49.2–57.8)	32.1 (28.2–36.3)	14.4 (11.6–17.7)
Increases my ability to do what is cost-effective for my patients	39.0 (34.8–43.2)	28.8 (25.0–32.9)	32.2 (28.4–36.5)
Reduces the role of insurance companies in the medication selection process	17.2 (14.3–20.8)	17.5 (14.5–21.0)	65.3 (61.2–69.4)
Forces me to take responsibility for a medication decision made by the insurance company	81.1 (77.5–84.3)	13.9 (11.1–17.1)	5.3 (3.6–7.5)
**Please indicate how much you agree or disagree with each of the following statements regarding the** **insurance process** **around a non-medical switch**^b^			
The process to challenge a non-medical switch is straightforward	17.3 (14.3–20.8)	15.0 (12.2–18.3)	66.9 (62.7–70.9)
The insurance process to challenge a non-medical switch is worth the effort	35.6 (31.6–39.8)	20.6 (17.3–24.3)	43.0 (36.1–44.6)
The insurance process discourages physicians from challenging a non-medical switch	76.7 (72.8–80.1)	14.4 (11.6–17.7)	8.2 (6.1–10.9)
Insurance companies provide clear steps to challenge a non-medical switch	20.4 (17.2–24.1)	20.0 (16.8–23.7)	58.9 (54.6–63.1)
Insurance companies clearly communicate how long it will take to receive a decision about a non-medical switch	17.9 (14.8–21.4)	18.0 (15.0–21.7)	63.2 (59.0–67.3)
Methods for inquiring about the status of a non-medical switch challenge are readily available	20.5 (17.2–24.1)	18.9 (15.7–22.5)	59.1 (54.8–63.3)
I am often put on hold for inconvenient lengths of time when I call insurance companies about my non-medical switch challenge	76.1 (72.2–79.6)	15.1 (12.3–18.5)	7.3 (5.3–9.8)
When I call an insurance company to challenge the non-medical switch, I spend most of my time speaking to a physician who has the requisite specialty or sub-specialty expertise to perform an expert review	19.7 (16.4–23.3)	15.5 (12.7–19.0)	62.0 (57.8–66.2)

^a^All percentages are weighted.

^b^Percentages may sum to less than 100% as respondents were allowed to indicate “don’t know” for this subset of survey items.

#### Impact on physician-patient relationship

More often than not, physicians agreed that non-medical switching diverted communication time away from other important clinical issues (84.3%), puts them in an uncomfortable situation where they are conflicted between the patient’s needs and the fiscal responsibilities of their practice (69.8%) and undermines a patient’s trust in their ability as a clinician (56.1%). Physicians were split in their opinion as to whether a non-medical switch increases their ability to do what is cost-effective for their patients.

#### Insurance process to challenge non-medical switches

When asked about how long the typical challenge process took, 55.9% of the physician sample reported that the process took between 1-week and 1-month (a median of 21 [0–126] days to complete the process from initiation of the challenge to a final decision). The mean success rate of non-medical switch challenges was reported to be 50.1%±21.6%.

Among physicians who were aware of the insurance process around a non-medical switch request (i.e., did not respond “don’t know”), most physicians disagreed that the process to challenge a non-medical switch is straightforward (66.9%), that insurance companies provide clear steps to challenge a non-medical switch (58.9%), that insurance companies clearly communicate how long it will take to receive a decision about a non-medical switch (63.2%), that methods for inquiring about the status of a non-medical switch challenge are readily available (59.1%) and that when they call an insurance company to challenge the non-medical switch, they spend most of their time speaking to a physician who has the requisite specialty or sub-specialty expertise to perform an expert review (62.0%). Conversely, most physicians agreed that the insurance process discourages physicians from challenging a non-medical switch (76.7%) and that they are often put on hold for inconvenient lengths-of-time when calling insurers about non-medical switch challenges (76.1%). Physicians’ opinions regarding whether the insurance process to challenge a non-medical switch is worth the effort was mixed, with 35.6% agreeing to some extent, 20.6% neither agreeing nor disagreeing and 43.0% disagreeing to some extent.

### Domain: Perceived impact on practice/staff and patients

#### Impact on practice/staff

Overall, 85.0% of respondents reported that the administrative workload of the practice increased to some extent, as did, the frequency of non-billable interactions with patients (72.5%) and the need for additional staffing requirements (62.2%). More than half (56.0%) of responding physicians reported that professional morale of their practice decreased (**Table 4**).

**Table 4:** Potential impact of non-medical switching on physicians, their office staff and patients.

How, if at all, does non-medical switch affect each of the following aspects of your practice?	% of Physicians Surveyed Responding “Increases Greatly”, “Increases Very Much” or “Increases Somewhat” (95%CI)^a^	% of Physicians Surveyed Responding “No Change” (95%CI)^a^	% of Physicians Surveyed Responding “Decreases Greatly”, “Decreases Very Much” or “Decreases Somewhat” (95%CI)^a^
Administrative work load of your practice	85.0 (81.7–87.8)	12.7 (10.0–15.8)	2.2 (1.3–4.0)
Frequency of non-billable interaction with patients	72.5 (68.6–76.2)	23.6 (20.1–27.4)	3.9 (2.5–5.9)
Additional staffing requirements	62.2 (58.0–66.3)	34.9 (31.0–39.2)	2.9 (1.8–4.8)
Professional morale	16.7 (13.8–20.2)	27.2 (23.6–31.2)	56.0 (51.7–60.3)
**What effect has non-medical switch had on your patients:**			
Effectiveness of treatment	14.1 (11.4–17.5)	39.4 (35.2–43.6)	46.5 (42.2–50.8)
Side effects	53.2 (48.8–57.4)	42.1 (38.0–46.5)	4.7 (3.2–6.9)
Medication adherence	14.2 (11.4–17.5)	35.2 (31.2–39.4)	50.6 (46.3–54.9)
Out-of-pocket medication costs	49.4 (45.1–53.7)	27.5 (23.9–31.6)	34.7 (30.6–38.8)
Abandonment of treatment	59.6 (55.2–63.7)	34.8 (30.8–39.0)	5.6 (4.0–8.0)
Frequency of medication errors	54.5 (50.2–58.7)	42.2 (38.0–46.5)	3.4 (2.1–5.2)

^a^All percentages are weighted.

#### Impact on patients

About forty-six percent of physicians indicated that they believed their patients experienced some degree of reduced efficacy. Between 49.5% and 59.6% of physicians reported they believed their patients experienced an increase in side effects, out-of-pocket costs, abandonment of treatment and medical errors as a result of non-medical switching; while ~50% indicated they thought patient adherence to their prescription medication decreased.

Some types of healthcare utilization were thought to more likely to occur as a result of non-medical switching; with 64.2% of physicians claiming non-office visit contacts and 69.5% claiming calls to/from the pharmacy were frequently increased (**Table 5**). Physician less frequently reported an increase in other types of healthcare utilization including office (33.6%) or emergency room visits (14.3%), laboratory tests (24.3%) and hospitalizations (13.1%). The need for additional medications was thought to increase less frequently (22.6%).

**Table 5:** Impact of non-medical switch on healthcare utilization.

How often does non-medical switch increase the frequency of each of the following?	% of Physicians Surveyed Responding “Very Frequently” or “Frequently” (95%CI)^a^	% of Physicians Surveyed Responding “Occasionally”, “Rarely” or “Never” (95%CI)^a^
Office visits	33.6 (29.7–37.9)	66.4 (62.1–70.3)
Non-office visit contacts (eg, phone, email)	64.2 (60.0–68.2)	35.8 (31.8–40.0)
Emergency room visits	14.3 (11.6–17.7)	85.7 (82.3–88.4)
Lab tests	24.3 (20.8–28.2)	75.7 (71.8–79.2)
Hospitalizations	13.1 (22.1–29.6)	86.9 (70.4–77.9)
Additional medications (for added effect or to manage side effects)	22.6 (19.2–26.4)	77.4 (73.6–80.8)
Calls to/from pharmacy	69.5 (65.3–73.3)	30.5 (26.7–34.5)

^a^All percentages are weighted.

## Discussion

The present, online, cross-sectional survey study of practicing physicians with experience with non-medical switching in prior 12-months provides 4 overarching findings. First, physicians are generally not fully open to requests for non-medical switching for many reasons, with physicians reporting patient’s stability on current medication as a critical factor influencing their decision to challenge a switch. Most physicians are either not open to (and will claim they would challenge a request) or have reservations regarding the switch. Despite physician reservations, non-medical switch requests are infrequently challenged; and when challenges occur, they appear as likely to fail as to succeed. Second, non-medical switching forces physicians to compromise their ethics as they frequently reported it undermined their autonomy and ability to treat patients in a cost-effective manner that is consistent with guidelines. Physicians also expressed concerns that non-medical switching negatively impacted patients’ trust in their care. Thirdly, physicians frequently reported that the practice of non-medical switching has a mostly negative impact on their practices and office morale. Most notably, non-medical switching required physicians to expend their staffs’ and own valuable time and resources to accommodate a switch request or navigate the complex challenge process. Finally, physicians felt non-medical switching negatively impacted patient care through increasing patient risk for side effects and medication errors, increasing patient out-of-pocket costs, increasing resource utilization and negatively influencing patient medication-taking behaviour (increasing medication abandonment, lowering adherence).

As the Quadruple Aim [3] emphasizes the partnership between patient and care team; the practice of non-medical switching threatens to disrupt the collaborative process of clinical decision-making between patient and physician. It has been previously demonstrated in a 2018 survey performed by the Alliance for the Adoption of Innovations of Medicine [10] that 57% of patients trust their physicians above all others when making treatment decisions, and overwhelmingly (87%) want insurers to play a secondary (or no) role in clinical decision-making, and yet, the practice of non-medical switching undermines this [17]. Interestingly, in that same survey, 65% of physicians reported concerns regarding “greater legal risks” due to insurer policies including non-medical switching [10].

Our study found decreased physician autonomy in practice and a loss of patient trust to be frequent consequences of non-medical switching, and these losses have been associated with physician burnout [18]. Physician burnout has been labelled a “public health crisis” that urgently requires action by healthcare institutions, governing bodies and regulatory authorities. Notably, the Centers for Medicare and Medicaid Services (CMS) and the American College of Physicians (ACP) have acknowledged that physicians are highly burdened by non-medical switching and other non-patient-care tasks and have subsequently launched burden reduction initiatives [4–5, 17]. It is estimated costs attributable to burnout are ~$4.6 billion per year in the US [19]. One recommended action to reduce physician burnout is reducing or streamlining insurance company processes [18].

Non-medical switching has been addressed by numerous stakeholders through guidance and position statements [1–2, 4–5, 8–13]. Most notably, the AMA strongly discourages non-medical switching for ambulatory patients with chronic diseases [1] and has provided recommendations to protect patients’ continuity-of-care, and ultimately improve patient health outcomes [2]. One such recommendation includes requiring that if a medication is removed from formulary after the beneficiary enrollment period is over, it should be covered for the duration of the benefit year to provide a buffer period for patients [2]. Multiple states have passed, and others have at least introduced, similar legislation [20].

Numerous studies have suggested a negative link between of non-medical switching and patient outcomes [6]. Nguyen and colleagues conducted a systematic review and identified 29 studies published between January 2000 and November 2015 that evaluated the impact of non-medical switching on health outcomes. Within identified studies, the impact of non-medical switching on a total of 96 separate health outcomes (60.4% clinical, 21.9% resource utilization, 13.5% economic, 4.2% medication-taking behaviour) was assessed. The investigators found that the effect of non-medical switching on outcomes was more frequently negative (33.3%) or neutral (55.2%) than positive (11.5%). In our survey, physician respondents highlighted their concern that non-medical switching can result in treatment inconsistent with accepted clinical guidelines and worsened outcomes in their patients. Moreover, our respondents’ assessment of increased healthcare utilization due to non-medical switching reflects a move in the wrong direction in this era of ever-increasing fiscal stewardship in healthcare.

While our study surveyed only physicians, a prior survey of patients to gain insight into patients’ impressions of non-medical switching has also been conducted [11]. In this survey of 143 patients performed by a special commission established by the Massachusetts Legislature; 70% of patient respondents reported experiencing decreased effectiveness, 86% reported worse side effects and 48% reported having to try multiple medications before finding an alternative that worked after a non-medical switch. Of note, nearly all of respondents (94%) stated they were in favor of legislation that would prohibit insurers from financially pressuring them to switch medications for non-medical reasons. Our physician respondents’ perceptions of the challenges facing their patients when presented with a non-medical switch mirror these patient responses.

Our study has several limitations worth discussing. First, as with any self-reported response survey, social desirability bias (whereby respondents answer questions in a manner that they feel will be viewed positively by others) may exist. Next, it is possible that both nonresponse bias [14–15, 21–22] and our sampling ratio of 60% primary care/40% specialist physicians may have impacted our survey’s results and their generalizability. Importantly, we achieved a reasonable response rate for a physician survey [14]. Moreover, we constructed study-specific post-stratification weights to adjust for nonresponse by physician type and differential sampling rates [15]. The latter required we estimate the proportion of active physicians in the US providing direct patient care that have a primary care (versus specialist) practice. While this data is available [16, 23–26], there is some variation in estimates across studies, with values ranging between 21.1% and 47.7% [23, 25]. We utilized a value of 30% for the proportion of physicians classifying themselves as primary care based upon the “2018 Physician Specialty Data Report” published by the Association of American Medical Colleges [16] which used the AMA’s 2018 Physician Masterfile (data as of December 31, 2017) as its primary data source. Thirdly, our study did not focus on a specific therapeutic indication and we cannot rule out that physicians’ opinions regarding non-medical switching varies by medication type. We did attempt to survey a broad set of physicians in this study including those practicing in different primary care settings as well as across multiple medical specialties. Fourth, to avoid respondent burden (and help achieve acceptable response rates), we used adaptive questioning, limited the number of questions in each domain and did not offer respondents the opportunity to provide an explanation or more nuanced response. Fifth, while we collapsed the 5- and 7-point Likert scales to improve interpretability of our survey’s results, this practice does come at the cost of lost information.

## Conclusions

Our study suggests physicians are often not open to or have reservations regarding insurance company requests for a non-medical switch. Non-medical switching results in a burden on physicians and their office staff. Requests for non-medical switches, the complexity of understanding and challenging a non-medical switch, as well as, associated ethical concerns appear to result in physician frustration. Many physicians believe they expend valuable time and resources addressing non-medical switch requests; which at least in part, may explain the infrequency in which such requests are challenged. Physicians also believe non-medical switching has some negative impacts on overall patient care, clinical outcomes and resource utilization. These data should be interpreted in context to important survey limitations including that these are physicians opinions (and association between non-medical switching and outcomes was not validated using real-world utilization data), response rate and generalizability to all US physicians as a group.

## Supporting information

S1 Fig Analytic framework describing the interplay between patients, physician practices, pharmacies and insurance companies due to a request for a non-medical switch of a prescription medication. The above analytic framework was developed to guide the discussion for the exploratory interviews used for survey development. During the exploratory interviews, a non-medical switch scenario and associated steps involving the patient, pharmacy, physician’s office and insurance were reviewed with physicians in order to understand the complete process to challenge a non-medical switch, factors impacting clinical decision-making, time and resources involved from physicians and their practice/ staff, insurance process and “interim” potential activities while the physician and patient was awaiting the challenge decision from the insurer. During the interviews, this analytic framework figure was broken out into 9 sections and color coded by stakeholder involved in each section.
(DOCX)

S1 Appendix Final survey. (DOCX)

S1 Table Additional survey response statistics. (DOCX)
