# Supplementary material for: Physicians’ perspectives regarding non-medical switching of prescription medications: Results of an internet e-survey
Source: PLoS One. 2020 Jan 10;15(1):e0225867. doi: 10.1371/journal.pone.0225867 (PMC6953849; doi:10.1371/journal.pone.0225867)
Supplement: S1 Fig — The above analytic framework was developed to guide the discussion for the exploratory interviews used for survey development. During the exploratory interviews, a non-medical switch scenario and associated steps involving the patient, pharmacy, physician’s office and insurance were reviewed with physicians in order to understand the complete process to challenge a non-medical switch, factors impacting clinical decision-making, time and resources involved from physicians and their practice/ staff, insurance process and “interim” potential activities while the physician and patient was awaiting the challenge decision from the insurer. During the interviews, this analytic framework figure was broken out into 9 sections and color coded by stakeholder involved in each section. (DOCX) [file pone.0225867.s001.docx]

**
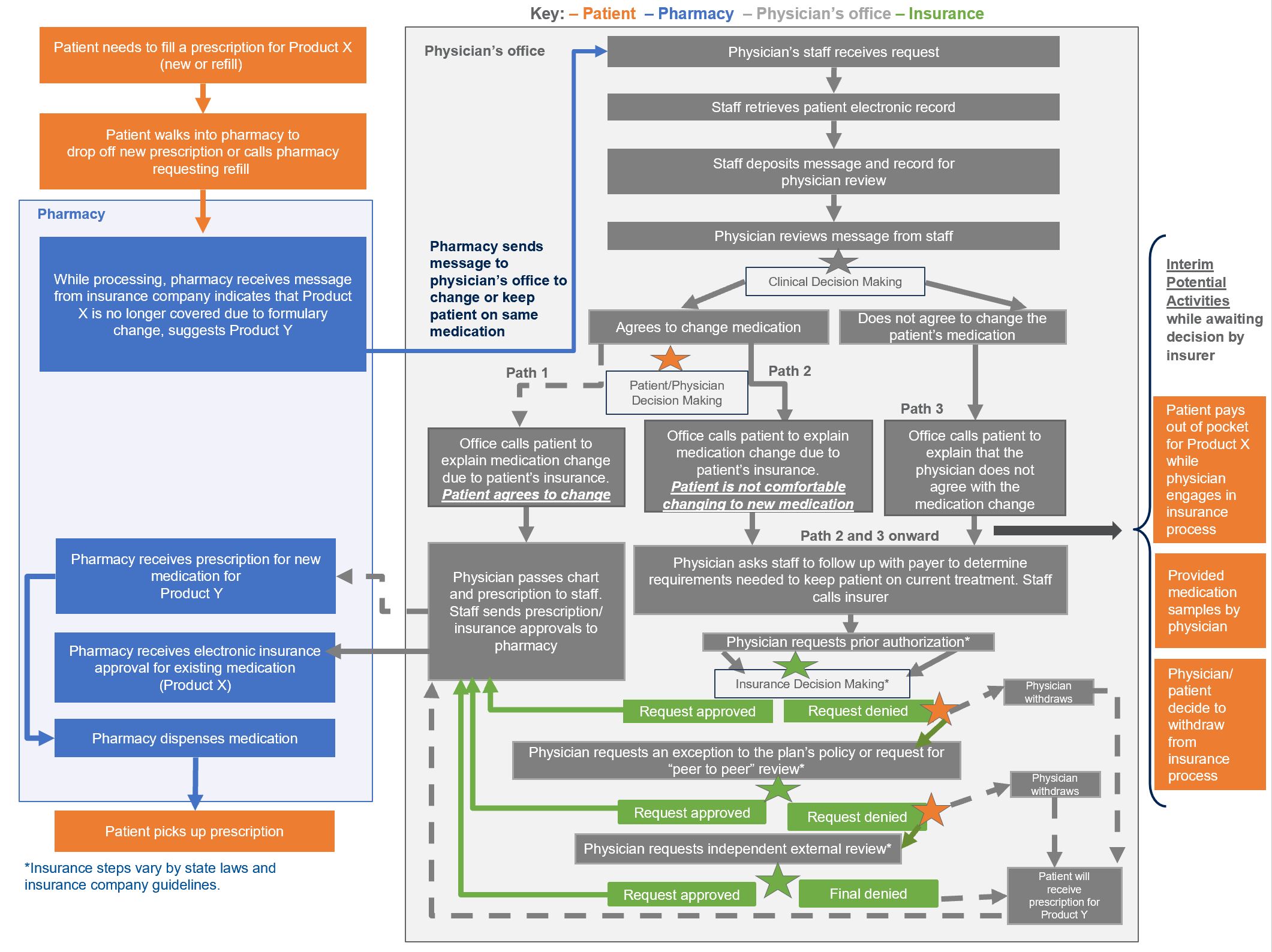
**

**eFigure 1. Analytic Framework Describing the Interplay Between Patients, Physician Practices, Pharmacies and Insurance Companies Due to a Request for a Non-Medical Switch of a Prescription Medication**

The above analytic framework was developed to guide the discussion for the exploratory interviews used for survey development. During the exploratory interviews, a non-medical switch scenario and associated steps involving the patient, pharmacy, physician’s office and insurance were reviewed with physicians in order to understand the complete process to challenge a non-medical switch, factors impacting clinical decision-making, time and resources involved from physicians and their practice/ staff, insurance process and “interim” potential activities while the physician and patient was awaiting the challenge decision from the insurer.
