# Supplementary material for: Physicians’ perspectives regarding non-medical switching of prescription medications: Results of an internet e-survey
Source: PLoS One. 2020 Jan 10;15(1):e0225867. doi: 10.1371/journal.pone.0225867 (PMC6953849; doi:10.1371/journal.pone.0225867)
Supplement: S1 Appendix — (DOCX) [file pone.0225867.s002.docx]

**eAppendix 1. Final Survey**

**We are conducting a survey among physicians. Please take 2 to 3 minutes to complete the screening questions in order to qualify for the survey. Your responses to these questions will be kept completely confidential.**

**S1. Which of the following best applies to you?**

1. General practice GO TO S3
2. Internal medicine GO TO S3
3. Family medicine GO TO S3
4. Specialist GO TO S2
5. Other (*Please specify*): _____________ TERMINATE

**S2. Which of the following is your primary medical specialty?**

1. Cardiology GO TO S3
2. Dermatology GO TO S3
3. Endocrinology GO TO S3
4. Gastroenterology GO TO S3
5. Oncology GO TO S3
6. Psychiatry GO TO S3
7. Rheumatology GO TO S3
8. Other (*Please specify*): _____________ TERMINATE

**S3. In which state do you practice? If you practice in more than 1 state, please choose where you manage the most patients.**

[DROP DOWN MENU OF ALL STATES IN THE US]

**S4. How many years have you been practicing/seeing patients post residency and/or fellowship?**

Number of years practicing/seeing patients TERMINATE IF ≤2 OR ≥30 YEARS

|  |  |  |
| --- | --- | --- |

**S5. What percentage of your professional time do you currently spend providing direct patient care?**

% of professional time spent providing direct patient care TERMINATE IF <10%

|  |  |  |
| --- | --- | --- |

Please consider the following standard definition below for **non-medical switching** before answering the next few questions:

**Non-medical switching is defined as a change in a stabilized patient’s prescribed medication to a chemically distinct alternative, not a generic, for reasons other than lack of clinical efficacy or response, side effects, or poor adherence.^1-3^**

Frequently, non-medical switching occurs due to formulary changes implemented by an insurance company.

1. Nguyen E, Weeda ER, Sobieraj DM, Bookhart BK, Piech CT, Coleman CI. Impact of non-medical switching on clinical and economic outcomes, resource utilization and medication-taking behavior: a systematic literature review. *Curr Med Res Opin.* 2016;32:7:1281-1290. DOI: 10.1185/03007995.2016.1170673
2. Gray T, Bertch K, Galt K, et al. ACCP position statement: guidelines for therapeutic interchange—2004. *Pharmacotherapy.* 2005;25:1666-1680.
3. H-125.991 Drug Formularies and Therapeutic Interchange. Chicago, IL: American Medical Association, 2016. <https://policysearch.ama-assn.org/policyfinder/detail/Drug%20Formularies%20and%20Therapeutic%20Interchange%20H-125.991?uri=%2FAMADoc%2FHOD.xml-0-227.xml>. Accessed August 6, 2018.

**S6. After reading the information [on the last two screens], do you now understand what is meant by non-**

**medical switch?**

□ Yes GO TO S7

□ No GO TO S6a

**S6b. Please read the description of non-medical switch again before continuing.**

□ Now I understand what you mean by non-medical switch GO TO S7

□ I still don’t understand. This survey is not for me TERMINATE

**S7. In the past 12 months, has your practice received a request for a non-medical switch for a patient?**

□ Yes GO TO S8

□ No TERMINATE

□ Not sure TERMINATE

**Thank you for answering these questions. Based on your responses, we would like to invite you to participate in a survey study. The survey will take approximately 20 minutes to complete, and you will receive an honorarium in exchange for your time and valued feedback.**

**All of your responses will remain completely confidential. The data from the survey will be blinded and results will only be presented in aggregate. As such, please make every effort to be open and honest when responding to the questions.**

**S8. Are you interested in participating?**

□ Yes GO TO A1

□ No TERMINATE

**To begin, ~~we~~ have a few questions about your clinical decision-making process related to non-medical switching over the last 12 months.**

**A1. When thinking about your decision regarding a non-medical switch, how frequently are you:**

|  | **Very Frequently** | **Frequently** | **Occasionally** | **Rarely** | **Never** |
| --- | --- | --- | --- | --- | --- |
| 1. Amenable to changing the medication without reservation?................................. | □5 | □4 | □3 | □2 | □1 |
| 1. Amenable to changing the medication with reservation?.......................... | □5 | □4 | □3 | □2 | □1 |
| 1. Not amenable to changing the medication, and contact the insurance company to challenge the non-medical switch?...................................................... | □5 | □4 | □3 | □2 | □1 |
| 1. Reluctant to change the medication, but do not challenge the non-medical switch due to the insurance process?................... | □5 | □4 | □3 | □2 | □1 |

**A2. How often do the following factors influence whether or not you are amenable to a non-medical switch request?**

|  | **Very Frequently** | **Frequently** | **Occasionally** | **Rarely** | **Never** |
| --- | --- | --- | --- | --- | --- |
| 1. Efficacy of the alternative medication…… | □5 | □4 | □3 | □2 | □1 |
| 1. Side effects of the alternative medication……………………………………….. | □5 | □4 | □3 | □2 | □1 |
| 1. Patient’s stability on current medication… | □5 | □4 | □3 | □2 | □1 |
| 1. Your prescribing experience with both the current and alternative medication……….. | □5 | □4 | □3 | □2 | □1 |
| 1. Patient concerns about alternative medication………………………………………… | □5 | □4 | □3 | □2 | □1 |
| 1. Patient requests a challenge to the non-medical switch…………………………………… | □5 | □4 | □3 | □2 | □1 |
| 1. Switch would be suboptimal to the patient……………………………………………… | □5 | □4 | □3 | □2 | □1 |
| 1. Administrative time required……………….. | □5 | □4 | □3 | □2 | □1 |
| 1. Narrow therapeutic window of the medication (eg, warfarin or Synthroid)…… | □5 | □4 | □3 | □2 | □1 |
| 1. Evidence-based medicine (EBM)/ guidelines………………………………………….. | □5 | □4 | □3 | □2 | □1 |
| 1. Frequency of dosing (eg, once daily vs. twice daily)………………………………………… | □5 | □4 | □3 | □2 | □1 |
| 1. Vulnerable populations (eg, minority, elderly, lower socioeconomic income)……. | □5 | □4 | □3 | □2 | □1 |
| 1. Your past experiences attempting to challenge a non-medical switch with insurance companies in general……………. | □5 | □4 | □3 | □2 | □1 |
| 1. Other (specify): ______............................. | □5 | □4 | □3 | □2 | □1 |

**A3. How often do you challenge a non-medical switch?**

|  | **Very Frequently** | **Frequently** | **Occasionally** | **Rarely** | **Never** |
| --- | --- | --- | --- | --- | --- |
| 1. On average, how often do you challenge a non-medical switch?................................. | □5 | □4 | □3 | □2 | □1 |

**A4. When you challenge a non-medical switch request, on average, how long does the process take (ie, from initiation of challenge to final decision)?**

□1 There is no lapse

□2 Less than 1 week

□3 Greater than 1 week to less than 1 month

□4 1 to 2 months

□5 3 to 6 months

**A5. When you challenge a non-medical switch, on average, what is your success rate?**

**Please move the slider to indicate your success rate in percent.**

**[Slider: 0% 100% ]**

**Now we have a few general questions about your opinion(s) regarding non-medical switching.**

**B1. Please indicate how much you agree or disagree with each of the following related to non-medical switching and your professional experience.**

|  | **Agree Strongly** | **Agree Very**  **Much** | **Agree Somewhat** | **Neither Agree Nor Disagree** | **Disagree Somewhat** | **Disagree Very**  **Much** | **Disagree Strongly** |
| --- | --- | --- | --- | --- | --- | --- | --- |
| 1. Frustrates me……………. | □7 | □6 | □5 | □4 | □3 | □2 | □1 |
| 1. Forces me to compromise my ethics… | □7 | □6 | □5 | □4 | □3 | □2 | □1 |
| 1. Puts me in an uncomfortable situation where I am conflicted between the patient’s needs and the fiscal responsibilities of my practice……................... | □7 | □6 | □5 | □4 | □3 | □2 | □1 |
| 1. Compromises my autonomy in clinical   decision making….……… | □7 | □6 | □5 | □4 | □3 | □2 | □1 |
| 1. Undermines my clinical judgment………………….. | □7 | □6 | □5 | □4 | □3 | □2 | □1 |
| 1. Diverts communication time with patients away from other important clinical issues………..…… | □7 | □6 | □5 | □4 | □3 | □2 | □1 |
| 1. Undermines a patient’s trust in my ability as a clinician……………………. | □7 | □6 | □5 | □4 | □3 | □2 | □1 |
| 1. Results in treatment inconsistent with accepted guidelines……. | □7 | □6 | □5 | □4 | □3 | □2 | □1 |
| 1. Increases my ability to do what is cost-effective for my patients………….. | □7 | □6 | □5 | □4 | □3 | □2 | □1 |
| 1. Reduces the role of insurance companies in the medication selection process………. | □7 | □6 | □5 | □4 | □3 | □2 | □1 |
| 1. Forces me to take responsibility for a medication decision made by the insurance company...................…. | □7 | □6 | □5 | □4 | □3 | □2 | □1 |

**B2. Please indicate how much you agree or disagree with each of the following statements regarding the insurance process around a non-medical switch.**

|  | **Agree Strongly** | **Agree Very**  **Much** | **Agree Somewhat** | **Neither Agree Nor Disagree** | **Disagree Somewhat** | **Disagree Very**  **Much** | **Disagree Strongly** |
| --- | --- | --- | --- | --- | --- | --- | --- |
| 1. The process to challenge a non-medical switch is straightforward……………… | □7 | □6 | □5 | □4 | □3 | □2 | □1 |
| 1. The insurance process to challenge a non-medical switch is worth the effort…………………............. | □7 | □6 | □5 | □4 | □3 | □2 | □1 |
| 1. The insurance process discourages physicians from challenging a non-medical switch…………..…... | □7 | □6 | □5 | □4 | □3 | □2 | □1 |
| 1. Insurance companies provide clear steps to challenge a non-medical   switch…………………………... | □7 | □6 | □5 | □4 | □3 | □2 | □1 |
| 1. Insurance companies clearly communicate how long it will take to receive a decision about a non-medical switch……………….. | □7 | □6 | □5 | □4 | □3 | □2 | □1 |
| 1. Methods for inquiring about the status of a non-medical switch challenge are readily available………… | □7 | □6 | □5 | □4 | □3 | □2 | □1 |
| 1. I am often put on hold for inconvenient lengths of time when I call insurance companies about my non-medical switch challenge………………………. | □7 | □6 | □5 | □4 | □3 | □2 | □1 |
| 1. When I call an insurance company to challenge the non-medical switch, I spend most of my time speaking to a physician who has the requisite specialty or sub-specialty expertise to perform an expert review…………………………… | □7 | □6 | □5 | □4 | □3 | □2 | □1 |

**Now we have a few questions about the potential impact of non-medical switch on you, your office staff and your patients.**

**C1. How, if at all, does non-medical switch affect each of the following aspects of your practice?**

|  | **Increases Greatly** | **Increases Very**  **Much** | **Increases Somewhat** | **No Change** | **Decreases Somewhat** | **Decreases Very**  **Much** | **Decreases Greatly** |
| --- | --- | --- | --- | --- | --- | --- | --- |
| 1. Administrative work load of your practice…… | □7 | □6 | □5 | □4 | □3 | □2 | □1 |
| 1. Frequency of non-billable interaction with patients………………… …. | □7 | □6 | □5 | □4 | □3 | □2 | □1 |
| 1. Additional staffing requirements…………….. | □7 | □6 | □5 | □4 | □3 | □2 | □1 |
| 1. Professional morale……. | □7 | □6 | □5 | □4 | □3 | □2 | □1 |

**C2. How often does non-medical switch increase the number of each of the following?**

|  | **Very Frequently** | **Frequently** | **Occasionally** | **Rarely** | **Never** |
| --- | --- | --- | --- | --- | --- |
| 1. Office visits……………………………………. …. | □5 | □4 | □3 | □2 | □1 |
| 1. Non-office visit contacts (eg, phone, email)………………………………………………. | □5 | □4 | □3 | □2 | □1 |
| 3. Emergency room visits………………………… | □5 | □4 | □3 | □2 | □1 |
| 4. Lab tests…………………………………………… | □5 | □4 | □3 | □2 | □1 |
| 1. Hospitalizations…………………………………. | □5 | □4 | □3 | □2 | □1 |
| 1. Additional medications (for added effect or to manage side effects)……………………. | □5 | □4 | □3 | □2 | □1 |
| 1. Calls to/from pharmacy……………………….. | □5 | □4 | □3 | □2 | □1 |

**C3. What effect has non-medical switch had on your patients:**

|  | **Increases Greatly** | **Increases Very**  **Much** | **Increases Somewhat** | **No Change** | **Decreases Somewhat** | **Decreases Very**  **Much** | **Decreases Greatly** |
| --- | --- | --- | --- | --- | --- | --- | --- |
| 1. Effectiveness of treatment.………………… | □7 | □6 | □5 | □4 | □3 | □2 | □1 |
| 1. Side effects……………….. | □7 | □6 | □5 | □4 | □3 | □2 | □1 |
| 1. Medication adherence… | □7 | □6 | □5 | □4 | □3 | □2 | □1 |
| 1. Out-of-pocket medication costs……..… | □7 | □6 | □5 | □4 | □3 | □2 | □1 |
| 1. Abandonment of treatment…………………. | □7 | □6 | □5 | □4 | □3 | □2 | □1 |
| 1. Frequency of medication errors………. | □7 | □6 | □5 | □4 | □3 | □2 | □1 |

**Now we have just a few final questions on your professional background.**

**D1. What is your gender?**

□1 Female

□2 Male

**D2. What is your age (in years)?**

|  |  |  |
| --- | --- | --- |

**D3. In which of the following practice settings do you see and treat patients? Select all that apply.**

□ Academic/teaching hospital

□ Outpatient centers (hospital-affiliated)

□ Community hospital

□ Private practice

□ Ambulatory surgical center

□ Other (specify): ___________

**D4. Approximately what percentage of your patients are covered as follows:**

______% Medicaid

______% Medicare/Medicare Advantage

______% Commercial

______% Uninsured and unable to pay

______% I do not know

**_100__% Total** *Must sum to 100%

**D5. In your main practice, what are the reimbursement mechanisms by which you are compensated for the care you provide to patients? Select all that apply.**

□ Fee-for-service

□ Value-based payment arrangements (ie, pay-for-performance, shared savings)

□ Salary

□ Other (specify): _____________
