# Supplementary material for: Physicians’ perspectives regarding non-medical switching of prescription medications: Results of an internet e-survey
Source: PLoS One. 2020 Jan 10;15(1):e0225867. doi: 10.1371/journal.pone.0225867 (PMC6953849; doi:10.1371/journal.pone.0225867)
Supplement: S1 Table — (DOCX) [file pone.0225867.s003.docx]

**S3 Table. Additional Survey Response Statistics**

| **Statistic** | **Total** |
| --- | --- |
| Click through | 1904 |
| Partial completion | 169 |
| Terminated | 570 |
| Over-quota (specialist physicians) | 155 |
| Qualified completion | 1010 |
| Completion rate | 85.7% |
| Completion time (mean; minutes:seconds) | 28:05 |
| Completion time (median; minutes:seconds) | 17:11 |
